# The U-shaped correlation between the systemic immune-inflammation index and all-cause and cardiovascular mortality in hyperlipidemic patients: findings from NHANES 1999–2018

**DOI:** 10.1080/07853890.2026.2656098

**Published:** 2026-04-13

**Authors:** Dongchi Ma, Qiongyu Zhou, Jiaying Yu, Chuyun Xu, Lili Yang, Li Ning

**Affiliations:** ^a^ School of Nursing, Zhejiang Chinese Medical University, Hangzhou, Zhejiang, China; ^b^ Department of Nursing, Affiliated Hangzhou First People’s Hospital, School of Medicine, Westlake University, Hangzhou, Zhejiang, China

**Keywords:** All-cause mortality, cardiovascular mortality, hyperlipidemia, NHANES, systemic immune-inflammation index, U-shaped

## Abstract

**Background:**

The relationship of systemic immune-inflammation index (SII) with mortality in hyperlipidemic patients remains unknown. In the present work, we evaluated SII for its ability to predict mortality incidence and examined its relationship with mortality of hyperlipidemic individuals.

**Methods:**

Altogether 27,903 hyperlipidemic cases were collected during 1999–2018 following the National Health and Nutrition Examination Survey (NHANES). SII was determined with complete blood count parameters. Our main results included all-cause and cardiovascular disease (CVD) mortality. We conducted Kaplan–Meier survival, restricted cubic spline (RCS), and Cox proportional hazards model analyses to assess the connection of SII to mortality among hyperlipidemic patients. To ensure that these results were reliable, we also conducted sensitivity and subgroup analyses.

**Results:**

For hyperlipidemic cases, their all-cause and CVD mortality rates were 12.651% and 3.916% separately. According to the Kaplan–Meier analysis, survival rates were low for individuals with high SII values. Multivariate Cox regression analysis indicated that for every one-unit elevation of lnSII, the all-cause mortality rate elevated by 19.7% (HR = 1.197, 95% CI: 1.117, 1.283), and the CVD mortality rate elevated by 30.1% (HR = 1.301, 95% CI: 1.131, 1.497). RCS suggested the U-shaped relationship of lnSII values with all-cause and CVD mortality (*p* < 0.0001) of hyperlipidemic individuals. According to our threshold analysis results, thresholds for lnSII were 6.08 and 5.96. Subgroup and sensitivity analyses yielded reliable results.

**Conclusions:**

For representative hyperlipidemic patients, SII shows the non-linear, U-shaped relationship with all-cause and CVD mortality.

## Introduction

Hyperlipidemia, as a systemic metabolic disorder, results from the aberrant increase in lipid components in the plasma beyond the normal physiological range, which includes elevated triglyceride, cholesterol, or low-density lipoprotein (LDL) contents but reduced high-density lipoprotein (HDL) contents [1,2]. The prevalence of hyperlipidemia is increasing globally [3]. Data from 2017 to 2020 showed that the total cholesterol content in about 86 million US adults exceeded 200 mg/dL [4]. The incidence of cardiovascular diseases (CVD) is increasing annually, and the mortality rate has increased from 12.1 to 18.6 million individuals from 1990 to 2019 [5]. Hyperlipidemia is an important CVD-related risk factor closely related to high mortality and also the main public health challenge that global disease prevention and control efforts strive to address [6].

Chronic inflammation influences blood lipid homeostasis and exacerbates the risk of fatal outcomes, particularly cardiovascular and non-cardiovascular outcomes [7,8]. The systemic immune-inflammation index (SII) combines three blood parameters to quantify systemic inflammation, and the value of this composite biomarker is obtained through the neutrophil–platelet product–lymphocyte ratio in the peripheral circulation [9]. The SII can effectively predict different disorders, including cancer [10,11], CVD [12], respiratory diseases [13], and psychiatric disorders [14]. The findings of meta-analyses have suggested a robust connection of SII to all-cause and CVD mortality [15]. A recent study reported that SII showed a non-linear association with atrial fibrillation recurrence after catheter ablation in hypertensive patients, supporting its potential value in cardiovascular outcome prediction [16]. In addition, Zhang et al. [17] demonstrated that systemic inflammatory indices, including SII, may contribute to mortality risk prediction and cardiovascular risk stratification in individuals with diabetes and prediabetes. Notably, studies have shown that SII is associated with hyperlipidemia and provides valuable information for predicting metabolic syndrome [18,19], indicating that inflammatory and metabolic pathways are closely intertwined. However, despite the established associations between SII and cardiovascular outcomes in general populations, and the recognized link between SII and lipid disorders, the specific relationship between SII and mortality outcomes in hyperlipidemic patients remains inadequately explored. Therefore, we aimed to investigate the association between SII and all-cause as well as cardiovascular mortality in a large nationally representative sample of hyperlipidemic adults in the United States.

The relation of SII with mortality risk was analyzed for hyperlipidemic subjects. By elucidating this relationship, we aimed to improve our understanding of how immunological and inflammatory responses affect the health outcomes of individuals with hyperlipidemia. Using the 1999–2018 National Health and Nutrition Examination Survey (NHANES) data, we comprehensively evaluated the relation of SII with mortality in this patient cohort. Our findings have important implications for improving prognostic techniques concerning the management of hyperlipidemia.

## Methods

### Data sources

The NHANES database is the nationwide survey created by the Centers for Disease Control and Prevention (CDC) for evaluating general health and nutritional status of the U.S. people. This comprehensive study used an exquisite multistage probability sampling methodology, and the National Center for Health Statistics Institutional Review Board approved our protocols. Before participating in this research, the individuals offered informed consent. A secondary analysis of deidentified NHANES data was performed for this study, which was exempted from further ethical approval because it presented negligible risk to participant confidentiality.

**Figure 1.**
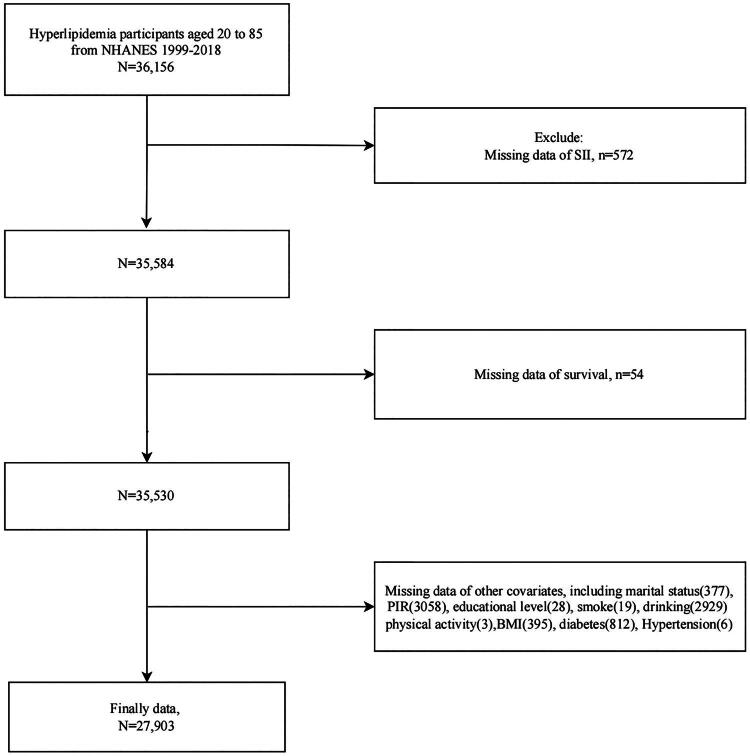
Participant selection and study flow diagram.

Individuals with hyperlipidemia surveyed in the NHANES database from 1999 to 2018 were considered to be the study population. The inclusion criteria included: (1) at least 20 years of age and (2) diagnosis of hyperlipidemia. Patients below were excluded: (1) missing data for SII indicators (platelet, neutrophil, and lymphocyte counts); (2) missing mortality data; (3) missing covariate data. Individuals with missing information, such as ‘NA’, ‘Don’t know’ or ‘other’, were excluded from this study. Ultimately, we enrolled 27,903 patients for analysis (Figure 1).

### Evaluation of hyperlipidemia

We used Adult Treatment Panel III standards from the National Cholesterol Education Program to evaluate hyperlipidemia [20]. If one or more of the following criteria were satisfied, hyperlipidemia was diagnosed: triglycerides ≥150 mg/dL, LDL ≥130 mg/dL, total cholesterol ≥200 mg/dL, or HDL ≤40 and ≤50 mg/dL in men and women, respectively [20]. Hyperlipidemia was also identified in patients who were using cholesterol-lowering agents [18].

### Calculation of the SII

SII were considered as exposure variables in this study. Full blood cell count (CBC) variables were obtained by Beckman Coulter method. The Coulter DxH 800 analyzer from the NHANES mobile laboratory performed complete blood counts, detailing the cellular distribution for all participants. Venous blood samples were collected by trained phlebotomists during the Mobile Examination Center (MEC) visit using EDTA tubes, and CBC assays were performed according to standardized NHANES laboratory protocols (fasting was not required for CBC testing) and analyzed within 24 h of collection. Lymphocyte, neutrophil, monocyte, and platelet counts were measured *via* complete blood count, and were presented as ×10^3^ cells/μL [21]. To be specific, SII was computed by: platelet number × neutrophil number/lymphocyte number [21]. Since the distribution of SII is specifically skewed to the right, SII values were natural log-transformed and grouped into four subgroups by its quartiles (*Q*1, *Q*2, *Q*3 and *Q*4), which were respectively included in models in the form of continuous and categorical variables. We used the first quartile of lnSII (*Q*1) as the reference group, and assigned a median value to each category to assess the linear trend.

### Evaluation of mortality

The primary endpoint of this study was all-cause mortality and the secondary endpoint was cardiovascular mortality. The National Death Index database created by the CDC was used to derive mortality statistics until December 31, 2019. The follow-up time was defined as the interval from the interview date to the date of mortality or through December 31, 2019 for alive participants. CVD mortality was examined according to the International Classification of Diseases, Tenth Revision (ICD-10) for classifying causes of death. The above classification was also used to identify cardiovascular causes of mortality, such as rheumatic heart disease (codes I00–I09), hypertensive heart and kidney illnesses (code I11), ischemic heart disease (code I13), heart failure (codes I20–I51), or cerebrovascular disorders (codes I60–I69). All-cause mortality refers to the total of all specific cause deaths.

### Evaluation of covariates

Based on previous studies [18], we included covariates in the analysis to take into account other factors confounding the results. This study covered the following covariates: age, sex, body mass index (BMI), race, education, marital status, poverty impact ratio (PIR), alcohol consumption, smoking, moderate physical activity, diabetes, hypertension, and CVD. The only continuous variable recorded was age. Gender had two categories, male and female. Among the races were non-Hispanic Black, non-Hispanic White, Mexican American, and other Hispanic. The education was classified as < high school, high school, or > high school level, whereas marital status was classified as single or non-single. Two PIR groups (≤3 and >3) were created. Three groups were created based on BMI: <25, 25–30, and ≥30. Three categories for smoking status were formed, which included never, currently, and previously. The categories of never, former, light, moderate, and excessive alcohol usage were identified. Three categories for physical activity were identified, which included vigorous, moderate, and none. CVD, diabetes, and hypertension were categorized as either yes or no. Multicollinearity was assessed using variance inflation factors (VIFs), with all VIFs below 5 (Table S1).

### Statistical analysis

To ensure that the estimates were nationally representative, suitable weights, stratification, and primary sample units were used to consider the intricate design of the NHANES. The data were compiled using descriptive techniques. Non-normally-distributed continuous data were represented by medians and interquartile ranges, while normally-distributed ones by mean and standard deviation (SD). Proportions were utilized for describing categorical data. The between-group differences in continuous data were determined through Student’s *t* tests, whereas those between categorical data were determined by the χ^2^ test.

The natural logarithm function was used to modify the skewed distribution of the SII in the analysis, and it was then split into quartiles. All subjects were classified in quartiles on the basis of lnSII values. The relationships of the ln-transformed SII with all-cause and CVD mortality in hyperlipidemic patients were assessed by multivariate weighted Cox regression models. There were three covariate-adjusted models: the crude model was not modified; Model 1 was adjusted for age, sex, race, PIR, education, and marital status; Model 2 was adjusted for smoking status, alcohol consumption, BMI, physical activity, diabetes, hypertension, and CVD along with all variables in Model 1. To compare survival outcomes across SII groups, we conducted Kaplan–Meier analysis for visualizing all-cause mortality and CVD mortality risks over time. We also conducted restricted cubic spline (RCS) analysis for assessing the dose–response relationship of mortality risk with log-transformed SII values. Analysis of the threshold effect was conducted for the non-linear relationship. Two-stage Cox proportional hazards regression analysis was also performed on two sides of the inflection point for examining relationships of ln-transformed SII with all-cause and CVD mortality.

To determine if the above factors altered the relation of SII with all-cause and CVD mortality risks, we conducted interaction tests and stratified analyses according to age, sex, BMI, race, PIR, marital status, education, smoking, alcohol consumption, physical activity, hypertension, diabetes, and CVD. Besides, sensitivity analyses included excluding data from patients that died in two years during follow-up, excluding participants with possible acute inflammation or infection (CRP >10 mg/L), and further adjusting for key medications and major baseline comorbidities to determine the validity of our conclusions. All test results were of statistical significance at *p* < 0.05. Analysis was completed with R version 4.2.3 software.

## Results

### Baseline features and participant demographics

The characteristics of the NHANES participants from 1999 to 2018 are shown in Table 1. According to our eligibility criteria, 27,903 hyperlipidemic individuals were enrolled (average age: 50.041 years), 48.913% of whom were men. The CVD and all-cause incidence rates were 3.916% and 12.651%, separately, and the median follow-up was 113 months. Based on their lnSII quartiles, participants with hyperlipidemia were divided equally into four groups: low lnSII (*Q*1), mid-low lnSII (*Q*2), mid-lnSII (*Q*3), and high lnSII (*Q*4). According to demographic analysis, the participants within the highest lnSII quartile (*Q*4) were probably older and comprised predominantly females, single individuals, and non-Hispanic White individuals; those having the high school education; past and current smokers; former and heavy drinkers; those with a high BMI; and those with a higher prevalence of diabetes, hypertension, and CVD. They were associated with the decreased vigorous physical activity proportion and lower PIR levels. In the aforementioned areas, the four groups differed significantly from one another.

**Table 1.** Baseline features of hyperlipidemia survivors according to lnSII quartile are shown.

Characteristic	Total (*N* = 27903)	*Q*1 (*N* = 6979)	*Q*2 (*N* = 6973)	*Q*3 (*N* = 6976)	*Q*4 (*N* = 6975)	*P* value
SII	566.770 ±3.182	264.112 ±0.963	414.889 ±0.582	571.105 ±0.771	969.176 ±4.749	<0.0001
Ln SII	6.208 ±0.006	5.536 ±0.005	6.024 ±0.001	6.343 ±0.001	6.828 ±0.004	<0.0001
Age (years)	50.041 ±0.193	50.377 ±0.285	49.518 ±0.259	49.721 ±0.283	50.584 ±0.294	0.001
PIR	3.059 ±0.030	3.069 ±0.039	3.125 ±0.037	3.101 ±0.037	2.943 ±0.042	0.0001
BMI	29.721 ±0.071	29.104 ±0.110	29.325 ±0.115	29.872 ±0.113	30.483 ±0.129	<0.0001
Gender, *n* (%)						<0.0001
Male	13797(48.913)	3734(53.471)	3524(51.199)	3381(48.613)	3158(43.088)	
Female	14106(51.087)	3245(46.529)	3449(48.801)	3595(51.387)	3817(56.912)	
Race, *n* (%)						<0.0001
Mexican American	4957(7.680)	1113(8.010)	1323(7.950)	1329(7.784)	1192(7.032)	
Non-Hispanic Black	4958(8.843)	2057(16.141)	1133(7.913)	932(6.482)	836(5.876)	
Non-Hispanic White	13533(72.475)	2602(63.966)	3288(72.357)	3652(74.996)	3991(77.341)	
Other Hispanic	2241(5.020)	567(4.998)	606(5.399)	569(5.163)	499(4.526)	
Other Race	2214(5.982)	640(6.885)	623(6.381)	494(5.576)	457(5.225)	
Marital status, *n* (%)						<0.0001
Non-single	17463(66.434)	4398(67.692)	4510(69.157)	4402(66.415)	4153(62.718)	
Single	10440(33.566)	2581(32.308)	2463(30.843)	2574(33.585)	2822(37.282)	
Education, *n* (%)						<0.001
<High school	3393(5.705)	869(6.019)	898(6.014)	837(5.441)	789(5.399)	
High school	10742(36.327)	2614(34.938)	2609(35.050)	2690(36.235)	2829(38.852)	
>High school	13768(57.968)	3496(59.042)	3466(58.937)	3449(58.324)	3357(55.750)	
Smoking status, *n* (%)						<0.0001
Never	14408(51.565)	3757(53.597)	3705(53.527)	3592(51.638)	3354(47.842)	
Former	7667(26.854)	1898(27.322)	1875(25.979)	1890(26.329)	2004(27.833)	
Now	5828(21.581)	1324(19.082)	1393(20.493)	1494(22.033)	1617(24.326)	
Alcohol consumption, *n* (%)						<0.001
Never	3986(11.241)	1054(12.356)	1008(11.429)	981(10.813)	943(10.532)	
Former	5427(16.195)	1342(15.297)	1283(14.942)	1310(16.084)	1492(18.297)	
Mild	9456(36.940)	2443(37.710)	2385(38.012)	2367(37.560)	2261(34.616)	
Moderate	3931(16.250)	961(16.210)	1021(16.603)	986(16.068)	963(16.121)	
Heavy	5103(19.374)	1179(18.427)	1276(19.014)	1332(19.474)	1316(20.434)	
Physical activity, *n* (%)						0.027
No	14859(47.151)	3795(47.928)	3653(46.536)	3687(46.232)	3724(48.005)	
Moderate	6899(27.319)	1626(26.028)	1774(27.489)	1709(27.219)	1790(28.356)	
Vigorous	6145(25.529)	1558(26.044)	1546(25.975)	1580(26.550)	1461(23.638)	
Diabetes, *n* (%)						0.02
No	22031(84.093)	5521(84.664)	5524(84.740)	5564(84.371)	5422(82.696)	
Yes	5872(15.907)	1458(15.336)	1449(15.260)	1412(15.629)	1553(17.304)	
Hypertension, *n* (%)						<0.0001
No	14360(56.611)	3619(57.847)	3794(59.556)	3604(56.655)	3343(52.634)	
Yes	13543(43.389)	3360(42.153)	3179(40.444)	3372(43.345)	3632(47.366)	
CVD, *n* (%)						<0.0001
No	24131(89.245)	6060(88.679)	6141(90.347)	6078(90.202)	5852(87.698)	
Yes	3772(10.755)	919(11.321)	832(9.653)	898(9.798)	1123(12.302)	

The results are represented by the mean (SD) or *n* (%) for categorical variables.

SII, systemic immune-inflammation; PIR, poverty income ratio; BMI, body mass index; CVD, cardiovascular disease.

### Kaplan–Meier survival analysis

**Figure 2.**
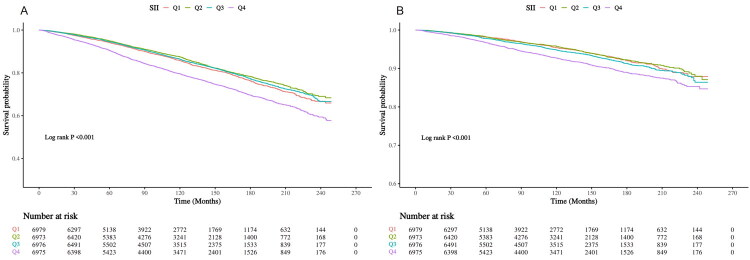
Kaplan–Meier analysis for all-cause mortality (A) and CVD-related mortality (B) across different SII groups.

Among the 27,903 participants with hyperlipidemia, 4,830 all-cause mortality, together with 1,579 CVD mortality cases were reported. Meanwhile, our Kaplan–Meier curves (Figure 2) revealed significant survival disparities across ln(SII) quartiles (*p* < 0.0001 for all-cause and CVD mortality), besides, the *Q*4 group showed accelerated mortality timing and increased event rates for both endpoints of the study. These results suggested that the SII substantially affects patient survival.

### Relationship of SII with all-cause and CVD mortality for hyperlipidemic patients

The SII was subjected to natural ln-transformation to standardize its distribution and improve the precision of the findings. Using three multivariate Cox regression models, the connections of lnSII to all-cause and CVD mortality of hyperlipidemic individuals were determined (Table 2). In individuals with hyperlipidemia, the lnSII had positive relation to all-cause and CVD mortality based on the crude model and Models 1 and 2, and this relationship was present even after adjusting for each confounder. The hazard ratio (HR) for all-cause mortality significantly increased to 1.197 (95% CI: 1.117, 1.283) for every unit increase in lnSII. When the adjusted HR was 1.175 (95% CI: 1.066, 1.296; *p* = 0.001), the largest SII quartile was strongly related to all-cause mortality versus the lowest counterpart.

**Table 2.** Relation of SII with mortality among hyperlipidemic patients.

	Crude model HR (95%CI)	*P* value	Model 1 HR (95%CI)	*P* value	Model 2 HR (95%CI)	*P* value
All-cause mortality						
Ln SII	1.309(1.200,1.426)	<0.0001	1.248(1.161,1.341)	<0.0001	1.197(1.117,1.283)	<0.0001
Quartile						
*Q*1	Reference		Reference		Reference	
*Q*2	0.825(0.732,0.929)	0.002	0.897(0.802,1.003)	0.057	0.861(0.770,0.962)	0.008
*Q*3	0.870(0.777,0.974)	0.015	0.923(0.834,1.021)	0.119	0.889(0.801,0.987)	0.028
*Q*4	1.282(1.150,1.428)	<0.0001	1.256(1.143,1.380)	<0.0001	1.175(1.066,1.296)	0.001
*P* for trend		<0.0001		<0.0001		<0.0001
CVD mortality						
Ln SII	1.443(1.242,1.677)	<0.0001	1.344(1.171,1.542)	<0.0001	1.301(1.131,1.497)	<0.001
Quartile						
*Q*1	Reference		Reference		Reference	
*Q*2	0.895(0.728,1.101)	0.294	0.999(0.828,1.206)	0.996	0.967(0.800,1.168)	0.725
*Q*3	0.960(0.802,1.148)	0.653	1.034(0.864,1.238)	0.714	1.006(0.835,1.212)	0.949
*Q*4	1.383(1.148,1.667)	<0.001	1.350(1.126,1.618)	0.001	1.277(1.056,1.544)	0.012
*P* for trend		<0.0001		<0.001		0.005

SII, systemic immune-inflammation; PIR, poverty income ratio; BMI, body mass index; CVD, cardiovascular disease; HR, hazard ratio; CI, confidence interval.

Crude Model: unadjusted.

Model 1: age, sex, race, PIR, education, and marital status.

Model 2: Model 1 + smoking status, alcohol consumption, physical activity, BMI, diabetes, hypertension, and CVD.

Similarly, when all covariates were adjusted, every unit increase in ln(SII) was related to the significantly elevated CVD mortality by 30.1% (HR = 1.301, 95% CI: 1.131, 1.497). As the categorical variable, the HRs and 95% CIs for mortality related to CVD in the upper lnSII quartiles were 0.967 (95% CI: 0.800, 1.168), 1.006 (95% CI: 0.835, 1.212), and 1.277 (95% CI: 1.056, 1.544), separately, relative to their lowest lnSII counterparts. Relative to *Q*1, the lnSII in *Q*4 was strongly associated with higher mortality related to CVD.

### Non-linear relationship of the SII with mortality

**Figure 3.**
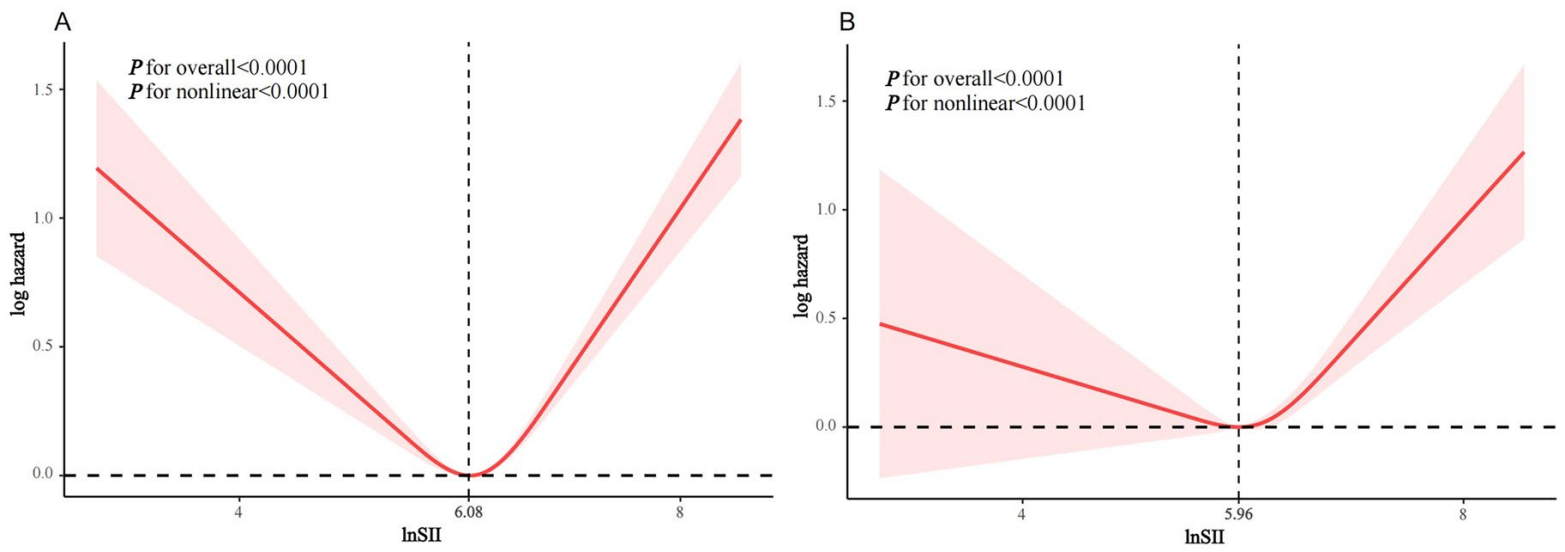
Relationships of lnSII with all-cause mortality (A) and CVD-related mortality (B) among hyperlipidemic patients are illustrated by the RCS.

Following full covariate adjustment, RCS regression suggested the non-linear relation of SII with mortality. The increasing SII values showed the U-shaped relationship with all-cause mortality risk (*p* < 0.0001 overall and non-linear) (Figure 3A). A similar U-shaped relationship could be detected in SII with CVD mortality risk (*p* < 0.0001 for overall and non-linear risk) (Figure 3B).

From Table 3, the piecewise Cox proportional hazards regression model was applied in better defining the relation of baseline lnSII with mortality because of the non-linear dose–response relationship of ln(SII) with mortality (all-cause and CVD). When the lnSII fell below the threshold value of 6.08, mortality risk was significantly and negatively correlated with lnSII for all-cause mortality (HR = 0.704, 95% CI: 0.614, 0.807). Additionally, all-cause mortality elevated by 1.702-fold per-unit elevation of lnSII when it was greater than 6.08 (HR = 1.702, 95% CI: 1.538, 1.882). When lnSII was above the threshold value of 5.96, a significant positive association was found for CVD mortality; each unit increase in lnSII increased CVD mortality risk by 1.669 times (HR = 1.669, 95% CI: 1.362, 2.046). However, when lnSII was less than 5.96, lnSII had no discernible effect on CVD-related mortality.

**Table 3.** Threshold effect of the SII on all-cause mortality and CVD mortality in hyperlipidemic patients.

Ln SII	All-cause mortality	*P* value	CVD mortality	*P* value
Threshold value	6.08		5.96	
lnSII ≤ Threshold value	0.704(0.614,0.807)	<0.0001	0.961(0.737,1.253)	0.767
lnSII > Threshold value	1.702(1.538,1.882)	<0.0001	1.669(1.362,2.046)	<0.0001
P-interaction	<0.0001		0.005	

Age, sex, race, PIR, education, marital status, smoking status, alcohol, physical activity, BMI, diabetes, hypertension, and CVD were adjusted for.

### Subgroup analysis

**Figure 4.**
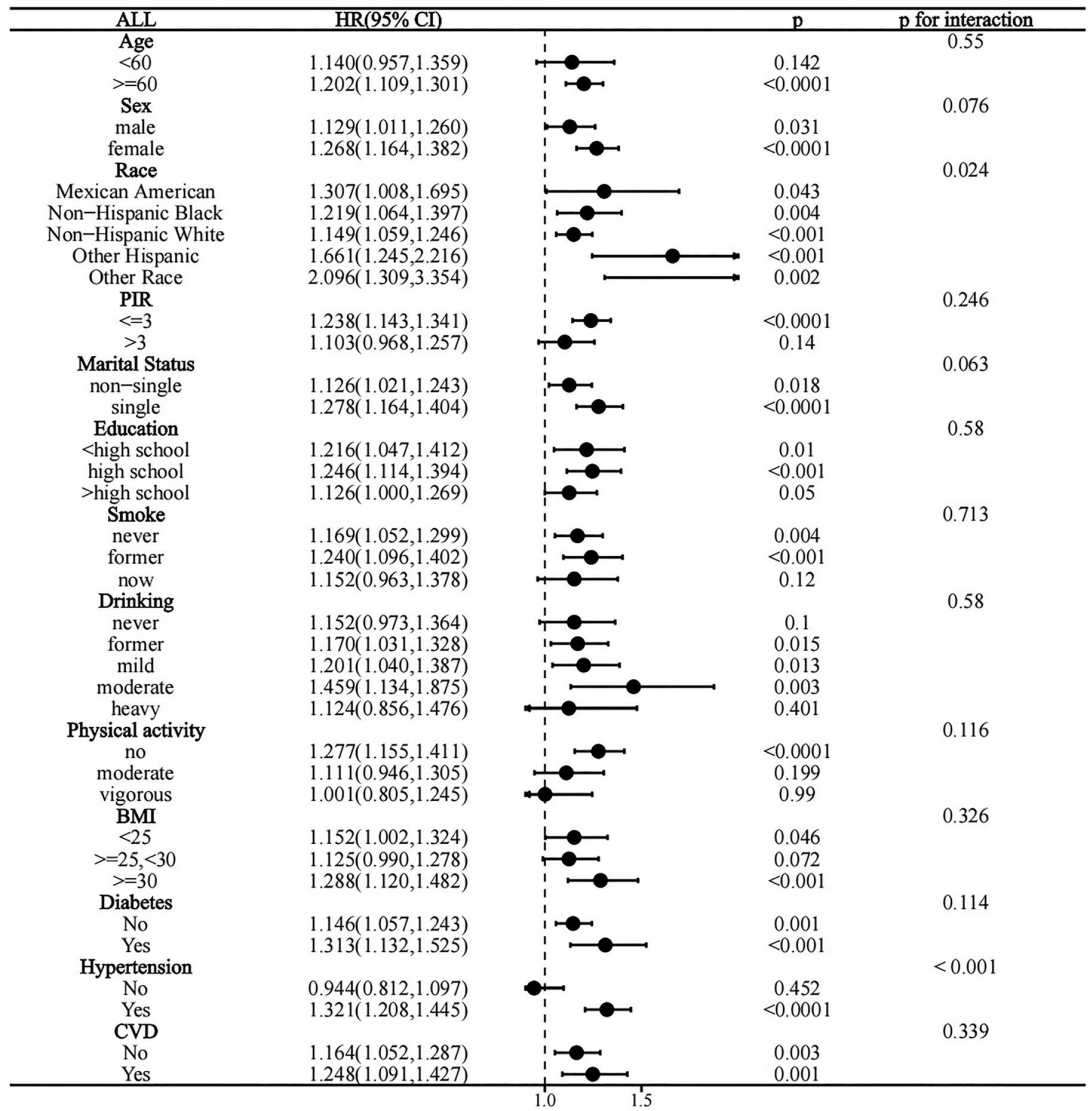
Forest plot of hazard ratios for all-cause mortality in hyperlipidemic patients.

**Figure 5.**
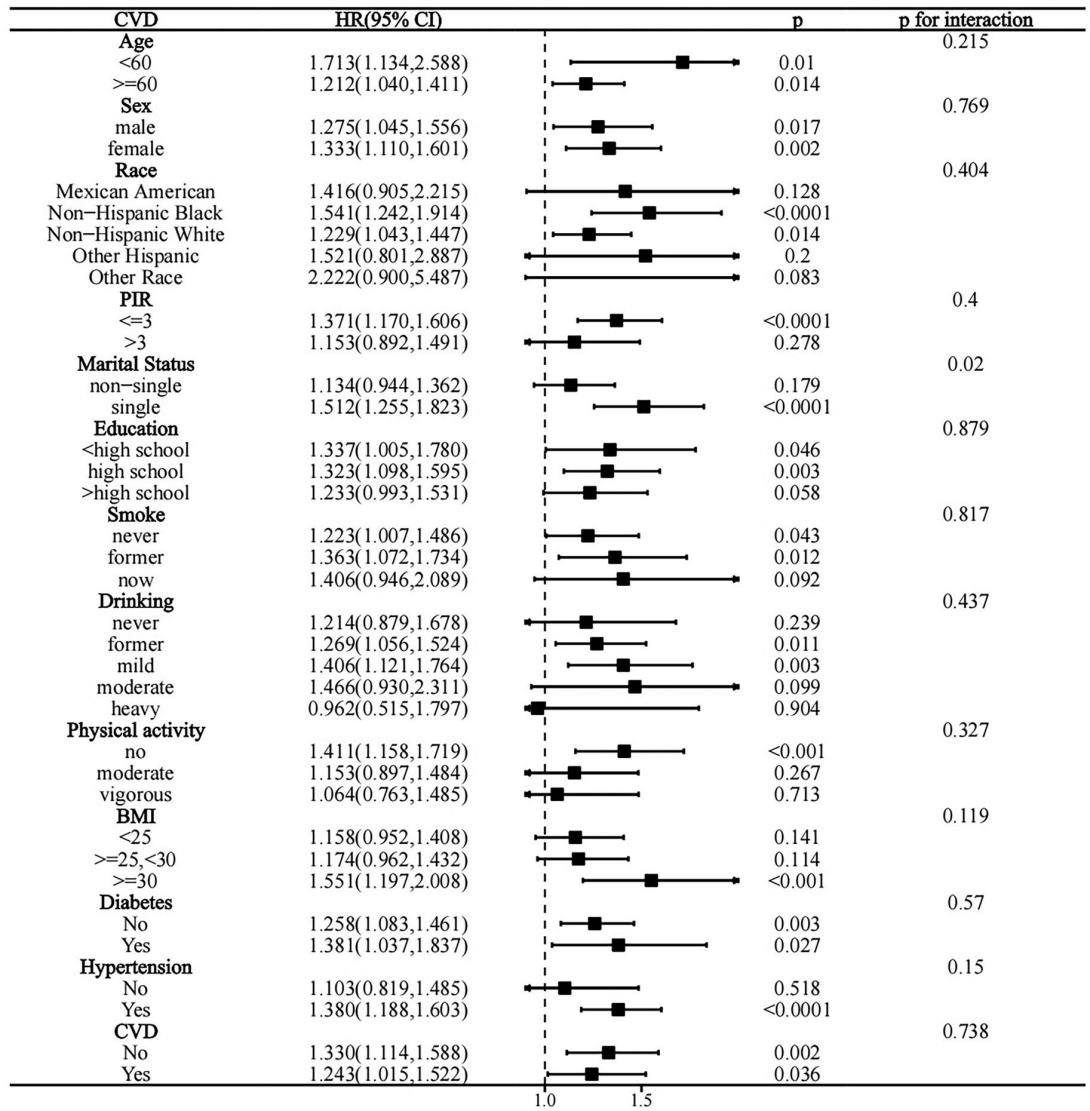
Forest plot of hazard ratios for CVD-related mortality in hyperlipidemic patients.

To better understand the relation of SII with all-cause and CVD-related mortality among individuals with hyperlipidemia, subgroup analyses were conducted (Figures 4 and 5). All subgroups exhibited the similar relationship of the lnSII with all-cause mortality (*P* for interaction >0.05). The SII showed stronger positive relation to all-cause mortality of hypertensive patients and all races (*P* for interaction <0.05).

The 1.521-fold increase in CVD mortality (95% CI: 1.225, 1.823) was related to the one-unit elevation of lnSII in the subgroup of single people. A one-unit elevation of lnSII was not strongly linked to the increased CVD mortality risk of the non-single-person category (HR = 1.134, 95% CI: 0.944, 1.362). Across all other stratification variables, no significant interactions of lnSII levels with CVD-related mortality were found (P for interaction > 0.05).

### Sensitivity analysis

A sensitivity analysis was conducted for confirming that our findings were robust. There were altogether 26,325 subjects enrolled into this work after those that died in two years during follow-up were eliminated. As shown in Supplementary Table 2, when all confounders were adjusted, the connection of SII with all-cause and CVD mortality remained unchanged. After excluding participants with potential acute infection (CRP > 10 mg/L), 25986 participants were included, and after adjusting for all confounding factors, the relationship between SII and all-cause and CVD mortality remained robust (Table S3). Additional adjustment for medication use and key comorbidities did not materially alter the results. In models further adjusted for infectives drugs use, anti-hyperlipidemic drugs use and key comorbidities, the association between SII and all-cause mortality remained significant, as did the association with CVD mortality (Table S4).

## Discussion

We examined the U-shaped relationship of SII with mortality outcomes for the large prospective cohort of hyperlipidemic individuals in the USA. The SII level, either low or high, was independently related to increased all-cause and CVD mortality risks in 27,903 hyperlipidemic patients. The ln-transformed SII values of 6.08 and 5.96, which translate to SII values of 435.35 and 391.52, respectively, were linked to lowest all-cause and CVD-related mortality risks. The greater SII value predicted the elevated all-cause and CVD-related mortality risks, according to the Kaplan–Meier analysis. These findings showed the utility of the SII as a low-cost, easily accessible marker to estimate the mortality of hyperlipidemia patients in clinical settings. Subgroup studies revealed possible relationships between SI’s accuracy in predicting all-cause mortality and race and hypertension; overall, the SII had a greater effect on CVD-related mortality in single individuals.

Genetic, metabolic, environmental, and other variables are the main causes of hyperlipidemia, a multifactorial illness with intricate pathophysiological pathways [22]. The development of hyperlipidemia is strongly influenced by inflammatory activities. In patients with hyperlipidemia, several peripheral blood inflammatory indicators serve as risk factors for negative outcomes. For instance, as reported by Ridker et al. [23], C-reactive protein was the reliable indicator of all-cause and CVD-related mortality for those on statin medication. However, few studies have investigated how the SII affects mortality outcomes in patients with hyperlipidemia. In this study, we investigated one of the initial efforts to systematically evaluate the relation of SII with all-cause and CVD-related mortality among the US hyperlipidemic people. An inflection point was identified, and the most important conclusion of the study was the non-linear, U-shaped relationship of the SII with all-cause as well as CVD-related mortality. In this study, given that SII can be easily calculated from routine blood tests, clinicians may incorporate this index into routine cardiovascular risk assessment to identify patients who may benefit from closer monitoring or anti-inflammatory strategies. In daily practice, SII values can inform specific clinical decisions including follow-up frequency, treatment intensity and specialist referral timing.

The relation of SII with mortality has been reported in various demographic groups in previous studies. In a study based on the US NHANES, greater SII values are linked to the increased all-cause risk by 29% (95% CI: 1.18–1.41) and the elevated CVD-related mortality risk by 22% (95% CI: 1.11–1.59) versus lower levels [24]. Zhang et al. [17] Found that SII is independent predictors of mortality in individuals with diabetes and prediabetes and established the value of systemic inflammation indices for cardiovascular risk stratification. Similar associations have also been confirmed in studies on mortality involving patients with osteoarthritis [25], breast cancer [26], diabetic nephropathy [27], and acute coronary syndrome [28]. However, studies focusing on hyperlipidemic populations are limited. Our study contributes to this growing body of evidence by specifically focusing on the hyperlipidemic population—a group with inherently elevated cardiovascular risk that has been underrepresented in previous inflammatory biomarker research. We found that in the adjusted model, one-unit elevation in lnSII was related to increases in all-cause and CVD-related mortality by 19.7% and 30.1%, respectively, with a U-shaped relationship. The U-shaped relationship often more accurately reflects the contribution of immune-inflammatory activity to disease development and has been reported in hypertension [12], rheumatoid arthritis [29], and sarcopenia [30]. This indicates that both excessively low and high levels of systemic inflammation may confer increased mortality risk, underscoring the importance of maintaining an optimal immune-inflammatory balance. However, the mechanism related to the U-shaped relationship is not fully understood. The SII related to the minimal mortality risk corresponds to the inflection point of this U-shaped association. When SII value is below this inflection point, the body is in a state of low inflammation. In this state, a moderate inflammatory response helps maintain immune surveillance functions [31], including promoting the clearance of oxidized lipids, maintaining endothelial homeostasis, and stabilizing plaques, all of which have protective effects [32]. Additionally, a balanced state of the immune system can support tissue repair while avoiding excessive inflammation that leads to endothelial damage, thereby reducing mortality risk in hyperlipidemia patients [33,34]. In contrast, when SII value is above the inflection point, it indicates a state of high inflammation, which may result in excessive release of pro-inflammatory factors. Neutrophil infiltration and platelet activation can exacerbate the risk of thrombosis and increase the likelihood of adverse outcomes in patients [35]. The U-shaped relationship we observed reveals that optimal immune-inflammatory balance is crucial, supporting personalized treatment approaches: patients with elevated SII (>435.35) warrant anti-inflammatory strategies including optimized statin therapy and lifestyle modifications, while those with very low SII (<391.52) require evaluation for potential immunosuppression or nutritional deficiencies. Clinicians could use this range to guide risk stratification and intensify follow-up for patients outside this window. However, these values should be interpreted cautiously and are not intended as validated clinical decision thresholds. Rather, they may serve as reference ranges for future research and hypothesis generation regarding optimal inflammatory states in hyperlipidemic populations. The accurate identification of residual cardiovascular risk in hyperlipidemia patients can be facilitated by the SII, which may guide personalized therapeutic strategies in clinical settings.

Extensive clinical studies have confirmed that the SII is a useful predictor of hyperlipidemic progression. A cross-sectional survey on an US population showed that a per-unit elevation of the SII is associated with the increased hyperlipidemia prevalence by 3% [16]. Correlations between lipid profiles and immune cell counts (neutrophils, lymphocytes, monocytes, and platelets) were identified in another study, indicating a relationship between the SII and lipid abnormalities [36]. Additionally, the SII was found to be positively related to LDL cholesterol in a cohort study conducted among adults in Saudi Arabia [37]. Zhao et al. [3] introduced various aspects of dyslipidemia while defining metabolic syndrome; as a result, the SII is negatively related to HDL content. These reports validated our finding that the SII serves as an autonomous risk marker for unfavorable clinical outcomes in individuals with high lipid levels.

Chronic low-grade inflammation serves as the critical factor exacerbating hyperlipidemia and CVD risks through interrelated biological mechanisms. Chronic inflammation induces hyperlipidemia by disrupting the balance among HDL, LDL, and triglycerides, resulting in dyslipidemia [38]. Although the SII is a marker reflecting inflammation, the mechanisms underlying its association with all-cause mortality in hyperlipidemic individuals need to be elucidated. Studies have suggested several potential pathways. Neutrophils promote vascular endothelial damage by releasing myeloperoxidase and neutrophil extracellular traps [39]. Neutrophils serve as key innate immune effector cells and release pro-inflammatory factors like IL-1β and IL-6, which intensify the systemic inflammatory state, directly participate in vascular endothelial damage and atherosclerotic plaque formation, and further exacerbate lipid deposition and vascular inflammatory responses [40,41]. The imbalance of lymphocyte subsets, particularly a decrease in regulatory T cells and an increase in Th17 cells, disrupts the anti-inflammatory/pro-inflammatory balance and accelerates atherosclerosis [42]. Activated platelets release mediators such as P-selectin and CD40L, which not only promote thrombosis but also activate vascular endothelial cells and monocytes, creating a vicious cycle of ‘inflammation–thrombosis’ [43]. The cumulative effect of these factors probably mediates the relation between SII and dismal prognostic outcome of hyperlipidemic patients, highlighting how the SII-quantified immune homeostasis influences disease progression.

Based on our subgroup analysis, among hyperlipidemic patients, the relation of SII with all-cause mortality was stronger in subgroups categorized based on race and hypertension status, whereas a similar relation of the SII with CVD mortality could be detected for subgroups classified based on marital status. The strength of the association of SII with all-cause mortality was significantly lower for the non-Hispanic white (HR = 1.149, 95% CI: 1.059–1.246) than for other populations, which may be associated with the high chronic inflammatory burden, metabolic disease burden, and disparities in access to healthcare resources among minority groups [44]. The coexistence of hypertension and hyperlipidemia exacerbates endothelial dysfunction and systemic inflammatory responses, thereby persistently activating the immune system and increasing the role of SII in predicting all-cause mortality risk [12]. Additionally, single individuals may experience an increased cardiovascular mortality risk due to factors including insufficient medical assistance and social support, higher smoking rates, poorer dietary habits, and lower participation in health screenings, making the connection of the SII to CVD-related mortality even more prominent among single hyperlipidemic patients [45,46]. Based on our sensitivity analysis, the connection of SII to all-cause and CVD mortality was stable.

We established systemic inflammatory markers as significant factors predicting all-cause and CVD mortality in hyperlipidemic subjects. Through demonstrating the clinical utility of these biomarkers, particularly the SII, we revealed their capacity to reflect immune activation and systemic inflammation. Our results suggested SII as the creditable prognostic indicator of hyperlipidemic outcomes, and helped understand the effect of inflammation on disease progression. From a practical standpoint, the SII offers several advantages for routine clinical application. Since it can be calculated from routine complete blood count results at no additional cost, SII assessment is readily implementable in daily practice. The identification of specific SII thresholds (435.35 for all-cause mortality and 391.52 for CVD mortality) provides actionable cutpoints that can guide clinical decision-making, enabling identification of higher-risk patients who may benefit from more intensive monitoring and personalized interventions. Furthermore, SII values can enhance patient–physician communication about prognosis and treatment adherence, with serial measurements providing tangible feedback on treatment effectiveness to improve patient engagement. Beyond individual patient management, SII could be integrated into existing cardiovascular risk prediction models to improve risk discrimination in hyperlipidemic populations, though future research is needed to validate this application. These insights may guide future diagnostic and therapeutic strategies, suggesting that integrating the SII with complementary biomarkers can increase the accuracy of risk assessment.

This study had several advantages. First, the NHANES database, a representative prospective cohort study in which follow-up was conducted for over 20 years, has advantages such as a large sample size and a sophisticated multistage probability sampling strategy. These features offer a strong foundation for deriving reliable findings with a significantly high statistical power. Second, this study analyzed the ability for SII to forecast mortality of dyslipidemic individuals and minimized potential impacts by correcting for numerous established risk factors. Furthermore, The SII is a simple, low-cost biomarker that can be easily derived from routine complete blood counts, making it highly accessible in both inpatient and outpatient settings. At last, for confirming the strength and dependability of the findings, comprehensive stratified and sensitivity analyses were conducted. The key clinical implication of our study is that both excessively low and high levels of systemic inflammation may increase mortality risk among hyperlipidemic patients. Monitoring inflammatory status using simple composite biomarkers such as SII may therefore help clinicians better identify high-risk individuals.

Some limitations must be noted in the present work. Since the NHANES is an observational study, it is impossible to confirm the causal relationship of SII with mortality. Second, the role of SII in death from other causes, such as cancer was not analyzed; instead, only the relation of SII with all-cause and CVD-related mortality was assessed. Third, only hyperlipidemic people in the US were included in the study, which restricted the generalizability and application of the model in other nations. To ensure that the results are applicable and universal, future studies should include multiple populations from different parts of the world. Fourth, the present analysis relied on NHANES cycles from 1999 to 2018 with mortality linkage available through December 31, 2019; therefore, more recent years, including the post-2020 period, could not be evaluated in this study. Future studies based on more contemporary cohorts are needed to determine whether the observed association remains stable in recent clinical settings. Finally, the SII might be probably affected by additional unknown factors, although the analysis corrected for several potential confounding factors. Even though our analysis identified an SII as a mortality inflection point, further studies are needed to determine how to apply this inflection point and whether it can be applied to other populations.

## Conclusion

To summarize, among adults with hyperlipidemia in the US, our results illustrated the U-shaped relationship of SII with all-cause and CVD-related mortality. Clinically, SII may serve as an easily obtainable adjunct marker to flag hyperlipidemic individuals who may benefit from intensified cardiovascular risk evaluation and follow-up when SII values are far below or above the nadir range. This implies that although the SII might be an effective indicator of survival, its relationship with mortality risk is complicated and needs further evaluation. These results showed that understanding and treating chronic inflammation in the hyperlipidemia setting is important, as this may be a viable strategy to decrease the risk of death and improve the prognosis for those who are affected. More investigation is warranted to confirm these relationships in multiple populations and investigate the therapeutic potential of modifying the SII.

## Additional of data and materials

Supplementary Material 1

## Declaration of generative AI

This study did not use AI-assisted writing.

## Supplementary Material

Supplementary_material_1-cleanversion.doc

## Abbreviations

BMI: Body mass index
CBC: Blood cell count
CDC: Centers for disease control and prevention
CI: Confidence interval
CVD: Cardiovascular disease
HDL: High-density lipoprotein
HR: Hazard ratio
LDL: Low-density lipoprotein
MEC: Mobile Examination Center
NHANES: National Health and Nutrition Examination Survey
PIR: Poverty impact ratio
RCS: Restricted cubic spline
SD: Standard deviations
SII: Systemic immune-inflammation index

## References

[1] Su X, Peng H, Chen X, et al. Hyperlipidemia and hypothyroidism. Clin Chim Acta. 2022;527:61–70. doi: 10.1016/j.cca.2022.01.006. 35038435

[2] Bozkurt B, Aguilar D, Deswal A, et al. Contributory risk and management of comorbidities of hypertension, obesity, diabetes mellitus, hyperlipidemia, and metabolic syndrome in chronic heart failure: a scientific statement from the American heart association. Circulation. 2016;134(23):e535–e78. doi: 10.1161/CIR.0000000000000450. 27799274

[3] Zhao ML, Zhang DW, Zhang QP, et al. Association between composite dietary antioxidant index and hyperlipidemia: a cross-sectional study from NHANES (2005–2020). Sci Rep. 2024;14(1):15935. doi: 10.1038/s41598-024-66922-0. 38987566 PMC11237065

[4] Tsao CW, Aday AW, Almarzooq ZI, et al. Heart disease and stroke statistics—2023 update: a report from the American heart association. Circulation. 2023;147(8):e93–e621. doi: 10.1161/CIR.0000000000001123. 36695182 PMC12135016

[5] Roth GA, Mensah GA, Johnson CO, et al. Global burden of cardiovascular diseases and risk factors, 1990–2019: update from the GBD 2019 study. J Am Coll Cardiol. 2020;76(25):2982–3021. doi: 10.1016/j.jacc.2020.11.010. 33309175 PMC7755038

[6] Mozaffarian D, Benjamin E, Go A, et al. Heart disease and stroke statistics − 2015 update: a report from the American heart association: a report from the American heart association. Circulation. 2015;131(4):e29-322–322. doi: 10.1161/CIR.0000000000000152. 25520374

[7] Van Diepen JA, Berbée JF, Havekes LM, et al. Interactions between inflammation and lipid metabolism: relevance for efficacy of anti-inflammatory drugs in the treatment of atherosclerosis. Atherosclerosis. 2013;228(2):306–315. doi: 10.1016/j.atherosclerosis.2013.02.028. 23518178

[8] Wang H, Nie HY, Bu G, et al. Systemic immune-inflammation index (SII) and the risk of all-cause, cardiovascular, and cardio-cerebrovascular mortality in the general population. Eur J Med Res. 2023;28(1):575. doi: 10.1186/s40001-023-01529-1. 38066657 PMC10709886

[9] Islam MM, Satici MO, Eroglu SE. Unraveling the clinical significance and prognostic value of the neutrophil-to-lymphocyte ratio, platelet-to-lymphocyte ratio, systemic immune-inflammation index, systemic inflammation response index, and delta neutrophil index: an extensive literature review. Turk J Emerg Med. 2024;24(1):8–19. doi: 10.4103/tjem.tjem_198_23. 38343523 PMC10852137

[10] Tian B-W, Yang Y-F, Yang C-C, et al. Systemic immune–inflammation index predicts prognosis of cancer immunotherapy: systemic review and meta-analysis. Immunotherapy. 2022;14(18):1481–1496. doi: 10.2217/imt-2022-0133. 36537255

[11] Ritter B, Greten FR. Modulating inflammation for cancer therapy. J Exp Med. 2019;216(6):1234–1243. doi: 10.1084/jem.20181739. 31023715 PMC6547855

[12] Cao Y, Li P, Zhang Y, et al. Association of systemic immune inflammatory index with all-cause and cause-specific mortality in hypertensive individuals: results from NHANES. Front Immunol. 2023;14:1087345. doi: 10.3389/fimmu.2023.1087345. 36817427 PMC9932782

[13] Luo ZB, Chen SY, Zhu N, et al. Relationship between systemic immune-inflammation index and long-term all-cause and cause-specific mortality among adult asthma patients: a population-based study. BMC Pulm Med. 2024;24(1):629. doi: 10.1186/s12890-024-03452-5. 39709369 PMC11663310

[14] Yu XP, Zheng HQ, Liu MX, et al. Association of systemic immune-inflammation index with all-cause and cardiovascular mortality among adults with depression: evidence from NHANES 2005–2018. BMC Psychiatry. 2025;25(1):25. doi: 10.1186/s12888-024-06463-y. 39780080 PMC11707943

[15] Li W, Wang X, Diao H, et al. Systemic immune inflammation index with all-cause and cause-specific mortality: a meta-analysis. Inflamm Res. 2024;73(12):2199–2216. doi: 10.1007/s00011-024-01959-5. 39400697

[16] Zhang Z, Li S, Tu T, et al. Nonlinear relationship and predictive value of systemic immune-inflammation index for atrial fibrillation recurrence after catheter ablation in hypertensive patients. Heart Rhythm. 2025;22(9):2257–2268. doi: 10.1016/j.hrthm.2025.03.1958. 40107395

[17] Zhang Z, Li C, Xiao Y, et al. Integrated machine learning and population attributable fraction analysis of systemic inflammatory indices for mortality risk prediction in diabetes and prediabetes. Ann Med. 2025;57(1):2536204. doi: 10.1080/07853890.2025.2536204. 40856552 PMC12302434

[18] Mahemuti N, Jing X, Zhang N, et al. Association between systemic immunity-inflammation index and hyperlipidemia: a population-based study from the NHANES (2015–2020). Nutrients. 2023;15(5):1177. doi: 10.3390/nu15051177. 36904176 PMC10004774

[19] Zeng P, Jiang C, Liu AB, et al. Association of systemic immunity–inflammation index with metabolic syndrome in us adult: a cross-sectional study. BMC Geriatr. 2024;24(1):61. doi: 10.1186/s12877-023-04635-1. 38225566 PMC10788994

[20] Lipsy RJ. The national cholesterol education program adult treatment panel iii guidelines. J Manag Care Pharm. 2003;9(1 Suppl):2–5. doi: 10.18553/jmcp.2003.9.s1.2. PMC10437161 14613351

[21] Hu B, Yang X-R, Xu Y, et al. Systemic immune-inflammation index predicts prognosis of patients after curative resection for hepatocellular carcinoma. Clin Cancer Res. 2014;20(23):6212–6222. doi: 10.1158/1078-0432.CCR-14-0442. 25271081

[22] Sudhakaran S, Bottiglieri T, Tecson KM, et al. Alteration of lipid metabolism in chronic kidney disease, the role of novel antihyperlipidemic agents, and future directions. Rev Cardiovasc Med. 2018;19(3):77–88. doi: 10.31083/j.rcm.2018.03.908. 31054556

[23] Ridker PM, Bhatt DL, Pradhan AD, et al. Inflammation and cholesterol as predictors of cardiovascular events among patients receiving statin therapy: a collaborative analysis of three randomised trials. Lancet. 2023;401(10384):1293–1301. doi: 10.1016/S0140-6736(23)00215-5. 36893777

[24] Xia YY, Xia CL, Wu LD, et al. Systemic immune inflammation index (SII), system inflammation response index (Siri) and risk of all-cause mortality and cardiovascular mortality: a 20-year follow-up cohort study of 42,875 US adults. J Clin Med. 2023;12(3):1128. doi: 10.3390/jcm12031128. 36769776 PMC9918056

[25] Zhou EY, Wu J, Zhou X, et al. Systemic inflammatory biomarkers are novel predictors of all-cause and cardiovascular mortality in individuals with osteoarthritis: a prospective cohort study using data from the nhanes. BMC Public Health. 2024;24(1):1586. doi: 10.1186/s12889-024-19105-5. 38872115 PMC11170786

[26] Li Y, Yu M, Yang M, et al. The association of systemic immune-inflammation index with incident breast cancer and all-cause mortality: evidence from a large population-based study. Front Immunol. 2025;16:1528690. doi: 10.3389/fimmu.2025.1528690. 39925802 PMC11802490

[27] Zhang F, Han Y, Mao YH, et al. The systemic immune-inflammation index and systemic inflammation response index are useful for predicting mortality in patients with diabetic nephropathy. Diabetol Metab Syndr. 2024;16(1):282. doi: 10.1186/s13098-024-01536-0. 39582034 PMC11587540

[28] Wang SP, Zhang GN. Association between systemic immune-inflammation index and adverse outcomes in patients with acute coronary syndrome: a meta-analysis. Angiology. 2025;76(10):946–954. doi: 10.1177/00033197241263399. 38904183

[29] Yin XS, Zhang Y, Zou JM, et al. Association of the systemic immune-inflammation index with all-cause and cardiovascular mortality in individuals with rheumatoid arthritis. Sci Rep. 2024;14(1):15129. doi: 10.1038/s41598-024-66152-4. 38956376 PMC11219888

[30] Guo B, Liu X, Si Q, et al. Associations of CBC-derived inflammatory indicators with sarcopenia and mortality in adults: evidence from NHANES 1999–2006. BMC Geriatr. 2024;24(1):432. doi: 10.1186/s12877-024-05012-2. 38755603 PMC11100216

[31] Andersen CJ. Lipid metabolism in inflammation and immune function. Nutrients. 2022;14(7):1414. doi: 10.3390/nu14071414. 35406026 PMC9002396

[32] Bender EC, Tareq HS, Suggs LJ. Inflammation: a matter of immune cell life and death. NPJ Biomed Innov. 2025;2(1):7. doi: 10.1038/s44385-025-00010-4. PMC13055066 42032111

[33] Theofilis P, Sagris M, Oikonomou E, et al. Inflammatory mechanisms contributing to endothelial dysfunction. Biomedicines. 2021;9(7):781. doi: 10.3390/biomedicines9070781. 34356845 PMC8301477

[34] Panchal SK, Brown L. Cholesterol versus inflammation as cause of chronic diseases. Nutrients. 2019;11(10):2332. doi: 10.3390/nu11102332. 31581553 PMC6835531

[35] Mcbride DA, Kerr MD, Dorn NC, et al. Triggers, timescales, and treatments for cytokine-mediated tissue damage. Euro Med J Innov. 2021;5(1):52–62. doi: 10.33590/emjinnov/20-00203. 34013158 PMC8130813

[36] Wei Y, Wang T, Li G, et al. Investigation of systemic immune-inflammation index, neutrophil/high-density lipoprotein ratio, lymphocyte/high-density lipoprotein ratio, and monocyte/high-density lipoprotein ratio as indicators of inflammation in patients with schizophrenia and bipolar disorder. Front Psychiatry. 2022;13:941728. doi: 10.3389/fpsyt.2022.941728. 35958647 PMC9360542

[37] Aljuraiban GS, Alharbi FJ, Aljohi AO, et al. Systemic immune-inflammation index and its relation to blood pressure and dyslipidemia in adults: a retrospective study. Medicine (Baltimore). 2024;103(28):e38810. doi: 10.1097/MD.0000000000038810. 38996174 PMC11245260

[38] Nepomuceno R, Pigossi SC, Finoti LS, et al. Serum lipid levels in patients with periodontal disease: a meta-analysis and meta-regression. J Clin Periodontol. 2017;44(12):1192–1207. doi: 10.1111/jcpe.12792. 28782128

[39] Smyth LCD, Murray HC, Hill M, et al. Neutrophil–vascular interactions drive myeloperoxidase accumulation in the brain in Alzheimer’s disease. Acta Neuropathol Commun. 2022;10(1):38. doi: 10.1186/s40478-022-01347-2. 35331340 PMC8944147

[40] Lin S, Zhu P, Jiang L, et al. Neutrophil extracellular traps induced by il-1β promote endothelial dysfunction and aggravate limb ischemia. Hypertens Res. 2024;47(6):1654–1667. doi: 10.1038/s41440-024-01661-3. 38605142

[41] Chen YQ, Mao RL, Chang Q, et al. A causal effects of neutrophil extracellular traps and its biomarkers on acute respiratory distress syndrome: a two-sample mendelian randomization study. Sci Rep. 2025;15(1):11995. doi: 10.1038/s41598-025-95676-6. 40199908 PMC11978891

[42] Picone F, Giudice V, Iside C, et al. Lymphocyte subset imbalance in cardiometabolic diseases: are t cells the missing link? Int J Mol Sci. 2025;26(3):868. doi: 10.3390/ijms26030868. 39940640 PMC11816853

[43] Bendas G, Gobec M, Schlesinger M. Modulating immune responses: the double-edged sword of platelet cd40l. Semin Thromb Hemost. 2025;51(8):855–869. doi: 10.1055/s-0044-1791512. 39379039

[44] Simons RL, Lei M-K, Klopack E, et al. Racial discrimination, inflammation, and chronic illness among African American women at midlife: support for the weathering perspective. J Racial Ethn Health Disparities. 2021;8(2):339–349. doi: 10.1007/s40615-020-00786-8. 32488825 PMC8183614

[45] Singh M, Nag A, Gupta L, et al. Impact of social support on cardiovascular risk prediction models: a systematic review. Cureus. 2023;15(9):e45836. doi: 10.7759/cureus.45836. 37881384 PMC10597590

[46] Klompstra L, Löf M, Björkelund C, et al. How are socioeconomic status, social support, and health history associated with unhealthy lifestyle behaviours in middle-aged adults? Results of the Swedish cardiopulmonary bioimage study (scapis) cohort. Arch Public Health. 2025;83(1):75. doi: 10.1186/s13690-025-01513-7. 40122851 PMC11931769

