# Supplementary material for: The U-shaped correlation between the systemic immune-inflammation index and all-cause and cardiovascular mortality in hyperlipidemic patients: findings from NHANES 1999–2018
Source: Ann Med. 2026 Apr 13;58(1):2656098. doi: 10.1080/07853890.2026.2656098 (PMC13078655; doi:10.1080/07853890.2026.2656098)
Supplement: Supplementary_material_1-cleanversion.doc [file IANN_A_2656098_SM2612.doc]

**Supplementary Table 1:** Variance Inflation Factors for Covariates

| **Variables** | **VIF** |
| --- | --- |
| lnSII | 1.013 |
| Age | 1.547 |
| Sex | 1.109 |
| Race | 1.098 |
| PIR | 1.324 |
| Education | 1.308 |
| Marital status | 1.083 |
| BMI | 1.131 |
| Alcohol consumption | 1.236 |
| Smoking status | 1.145 |
| Physical activity | 1.081 |
| Hypertension | 1.334 |
| Diabetes | 1.176 |
| CVD | 1.168 |

This table presents the VIFs for the covariates included in the models for both the NHANES.

**Abbreviation:** BMI: body mass index. CVD:Cardiovascular disease. PIR:Poverty impact ratio. SII:Systemic immune-inflammation index. VIFs: Variance Inflation Factors.

**Supplementary Table 2: The association between the SII with the all-cause and CVD mortality of the hyperlipidemia population (Excluding participants who died within two years of follow up in NHANES 1999–2018).**

|  | **Crude Model**  **HR (95%CI)** | **P-value** | **Model 1**  **HR (95%CI)** | **P-value** | **Model 2**  **HR (95%CI)** | **P-value** |
| --- | --- | --- | --- | --- | --- | --- |
| **All-cause mortality** | | | | | | |
| Ln SII | 1.232(1.127,1.346) | <0.0001 | 1.194(1.107,1.288) | <0.0001 | 1.149(1.066,1.238) | <0.001 |
| Quartile | | | | | | |
| Q1 | Reference |  | Reference |  | Reference |  |
| Q2 | 0.817(0.729,0.916) | <0.001 | 0.893(0.797,1.002) | 0.053 | 0.860(0.768,0.962) | 0.008 |
| Q3 | 0.861(0.767,0.966) | 0.011 | 0.918(0.821,1.027) | 0.134 | 0.888(0.792,0.996) | 0.043 |
| Q4 | 1.221(1.093,1.363) | <0.001 | 1.212(1.094,1.343) | <0.001 | 1.141(1.027,1.267) | 0.014 |
| P for trend |  | <0.0001 |  | <0.0001 |  | <0.001 |
| **CVD mortality** | | | | | | |
| Ln (SII) | 1.344(1.152,1.569) | <0.001 | 1.276(1.107,1.471) | <0.001 | 1.240(1.071,1.436) | 0.004 |
| Quartile | | | | | | |
| Q1 | Reference |  | Reference |  | Reference |  |
| Q2 | 0.872(0.705,1.078) | 0.206 | 0.984(0.805,1.202) | 0.873 | 0.955(0.781,1.169) | 0.656 |
| Q3 | 0.934(0.787,1.109) | 0.435 | 1.013(0.839,1.223) | 0.893 | 0.991(0.818,1.201) | 0.929 |
| Q4 | 1.325(1.091,1.610) | 0.005 | 1.314(1.086,1.589) | 0.005 | 1.250(1.024,1.526) | 0.028 |
| P for trend |  | <0.001 |  | 0.002 |  | 0.012 |

Abbreviations SII, Systemic Immune-Inflammation; PIR, poverty income ratio; BMI, body mass index; CVD, cardiovascular disease; HR, hazard ratio; CI, confidence interval

Crude Model: No-adjust

Model 1: Age, gender, race, PIR, educational level, marital statys

Model 2: Age, gender, race, PIR, educational level, marital status, Smoking status, Alcouol, physical activity, BMI, diabetes, Hypertension, CVD

**Supplementary Table 3: The association between the SII with the all-cause and CVD mortality of the hyperlipidemia population. (Excluding the participants with acute infection at baseline).**

|  | **Crude Model**  **HR (95%CI)** | **P-value** | **Model 1**  **HR (95%CI)** | **P-value** | **Model 2**  **HR (95%CI)** | **P-value** |
| --- | --- | --- | --- | --- | --- | --- |
| **All-cause mortality** | | | | | | |
| Ln SII | 1.296(1.188,1.414) | <0.0001 | 1.201(1.115,1.293) | <0.0001 | 1.160(1.078,1.248) | <0.0001 |
| Quartile | | | | | | |
| Q1 | Reference |  | Reference |  | Reference |  |
| Q2 | 0.847(0.743,0.965) | 0.013 | 0.924(0.819,1.043) | 0.201 | 0.890(0.793,1.000) | 0.050 |
| Q3 | 0.862(0.768,0.966) | 0.011 | 0.891(0.801,0.991) | 0.033 | 0.862(0.775,0.959) | 0.007 |
| Q4 | 1.287(1.155,1.435) | <0.0001 | 1.215(1.103,1.339) | <0.0001 | 1.145(1.037,1.263) | 0.007 |
| P for trend |  | <0.0001 |  | <0.0001 |  | 0.001 |
| **CVD mortality** | | | | | | |
| Ln (SII) | 1.435(1.214,1.697) | <0.0001 | 1.286(1.108,1.493) | <0.001 | 1.254(1.078,1.459) | 0.003 |
| Quartile | | | | | | |
| Q1 | Reference |  | Reference |  | Reference |  |
| Q2 | 0.878(0.712,1.082) | 0.222 | 0.981(0.812,1.184) | 0.839 | 0.900(0.748,1.083) | 0.265 |
| Q3 | 0.930(0.765,1.130) | 0.464 | 0.972(0.799,1.182) | 0.774 | 0.906(0.746,1.100) | 0.320 |
| Q4 | 1.344(1.094,1.650) | 0.005 | 1.248(1.024,1.522) | 0.028 | 1.108(1.004,1.358) | 0.024 |
| P for trend |  | 0.001 |  | 0.021 |  | 0.025 |

Crude Model: No-adjust

Model 1: Age, sex, race, PIR, educational level, marital

Model 2: Age, sex, race, PIR, educational level, marital, Smoke, Drinking, Activity, BMI, DM, Hypertension, CVD

**Supplementary Table 4:** The association between the SII with the all-cause and CVD mortality of the hyperlipidemia population adjuested for medication use and key comorbidities

|  | **Crude Model**  **HR (95%CI)** | **P-value** | **Model 1**  **HR (95%CI)** | **P-value** | **Model 2**  **HR (95%CI)** | **P-value** | **Model 3**  **HR (95%CI)** | **P-value** |
| --- | --- | --- | --- | --- | --- | --- | --- | --- |
| **All-cause mortality** | | | | | | |  |  |
| Ln SII | 1.311(1.201,1.431) | <0.0001 | 1.249(1.161,1.344) | <0.0001 | 1.190(1.110,1.276) | <0.0001 | 1.151(1.072,1.235) | <0.001 |
| Quartile | | | | | | |  |  |
| Q1 | Reference |  | Reference |  | Reference |  |  |  |
| Q2 | 0.825(0.733,0.928) | 0.001 | 0.888(0.793,0.994) | 0.040 | 0.861(0.771,0.963) | 0.008 | 0.836(0.745,0.938) | 0.002 |
| Q3 | 0.873(0.780,0.977) | 0.018 | 0.918(0.830,1.016) | 0.099 | 0.884(0.797,0.981) | 0.020 | 0.861(0.777,0.954) | 0.004 |
| Q4 | 1.281(1.150,1.427) | <0.0001 | 1.251(1.138,1.375) | <0.0001 | 1.170(1.062,1.290) | 0.002 | 1.113(1.007,1.231) | 0.035 |
| P for trend | | <0.0001 |  | <0.0001 |  | <0.0001 |  | 0.001 |
| **CVD mortality** | | | | | | |  |  |
| Ln (SII) | 1.446(1.242,1.684) | <0.0001 | 1.344(1.172,1.542) | <0.0001 | 1.301(1.131,1.496) | <0.001 | 1.245(1.081,1.433) | 0.002 |
| Quartile | | | | | | |  |  |
| Q1 | Reference |  | Reference |  | Reference |  |  |  |
| Q2 | 0.893(0.725,1.100) | 0.288 | 0.984(0.814,1.190) | 0.869 | 0.965(0.798,1.166) | 0.709 | 0.918(0.759,1.111) | 0.379 |
| Q3 | 0.964(0.804,1.156) | 0.692 | 1.027(0.857,1.231) | 0.770 | 1.000(0.829,1.205) | 0.998 | 0.962(0.801,1.155) | 0.680 |
| Q4 | 1.382(1.143,1.670) | <0.001 | 1.341(1.117,1.609) | 0.002 | 1.275(1.055,1.540) | 0.012 | 1.190(1.080,1.445) | 0.039 |
| P for trend | | <0.0001 |  | <0.001 |  | 0.005 |  | 0.030 |

Crude Model: No-adjust

Model 1:Age, sex, race, PIR, educational level, marital

Model 2: Age, sex, race, PIR, educational level, marital, Smoke, Drinking, Activity, BMI, DM, Hypertension, CVD, infectives drugs and anti Hyperlipidemic drugs

Model 3: Age, sex, race, PIR, educational level, marital, Smoke, Drinking, Activity, BMI, DM, Hypertension, CVD, infectives drugs and anti Hyperlipidemic drugs, Chronic kidney disease
